# Azacitidine-induced cryptogenic organizing pneumonia: a case report and review of the literature

**DOI:** 10.1186/s13256-016-0803-0

**Published:** 2016-01-20

**Authors:** Yanal Alnimer, Samer Salah, Bashar Abuqayas, Kamal Alrabi

**Affiliations:** 1 Internal Medicine Department, Hurley Medical Center, Flint, MI USA; 2 Medical Oncology Department, King Hussein Cancer Center, Amman, Jordan; 3 Internal Medicine Department, Trinitas Regional Medical Center – Seton Hall University School of Health and Medical Sciences, Elizabeth, NJ USA

**Keywords:** Azacitidine, Hypomethylating, Myelodysplasia syndrome, Organizing pneumonia

## Abstract

**Background:**

Myelodysplasia syndrome is a heterogeneous group of hematological disorders that are characterized by abnormal morphology and cytopenias of bone marrow elements. Azacitidine is a hypomethylating agent that is commonly used in treatment of myelodysplasia syndrome. We present an extremely rare case of cryptogenic organizing pneumonia following therapy with azacitidine and a review of the relevant literature. This is the fifth case of azacitidine-induced interstitial lung disease and the sixth one due to hypomethylating drugs; of interest, this is the first reported case that has occurred after the second cycle. Our case report highlights an important, potentially treatable and rare side effect of azacitidine and hypomethylating agents in general that might be overlooked by oncologists. Furthermore, our review of the literature showed heterogeneity in the clinical outcome which might, in part, be due to delay in initiating corticosteroids treatment.

**Case presentation:**

A 67-year-old white man presented with worsening shortness of breath and mild productive cough that started 1 week prior to his presentation. An initial chest X-ray showed infiltration of both lung fields. Radiographic findings of computed axial tomography, results of bronchoscopy and a lung biopsy were consistent with cryptogenic organizing pneumonia. The patient showed variable clinical response to steroids and he remained dependent on home oxygen.

**Conclusions:**

We concluded that there is a recognizable potentially life-threatening toxicity due to organizing pneumonia secondary to azacitidine in the setting of myelodysplasia syndrome treatment. This toxicity is not limited to the first cycle as in previous cases; furthermore, pleural effusion can be associated with this toxicity. Health care professionals should be aware of this recognizable side effect. Early recognition and timely management are critical to prevent permanent lung fibrosis.

## Background

Myelodysplasia syndrome (MDS) is a heterogeneous group of hematological disorders, which is broadly characterized by cytopenias, dysmorphic (or abnormally appearing) cellular elements of the bone marrow, and by consequent ineffective blood cell production. MDS is mostly a disease of older patients with median age at diagnosis of ≥65 years, and is characterized by male predominance [1]. Although onset of the disease earlier than 50 years of age is unusual, therapy-related MDS is one of the common etiologies in this age group [2]. Patients with MDS can present with a wide spectrum of presentations; however, signs and symptoms are usually nonspecific. Some of the commonly encountered presenting symptoms include fatigue, weakness and recurrent infections, whereas other cases are diagnosed incidentally due to cytopenias in routine blood tests.

Azacitidine is a pyrimidine nucleoside analog that has been used in treatment of MDS. The mechanism of action involves hypomethylation of DNA that results in increased expression of multiple genes leading to enhanced cellular maturation [3]. In this case report, we intend to report a case of azacitidine-induced interstitial lung disease (ILD) and to present a review of the relevant literature.

We were able to identify four similar reported cases following azacitidine treatment for MDS. Hematologists, oncologists, and health care professionals should be aware of the rare but potentially life-threatening toxicity of azacitidine.

## Case presentation

A 67-year-old white man presented with worsening shortness of breath and mild productive cough that started approximately 1 week prior to his presentation. His past medical history was remarkable for type II diabetes, hypertension and MDS with excess blast II, with some fibrosis in his bone marrow, for which he received two cycles of 5-azacitidine, with the last cycle delivered 2 weeks prior to his presentation. His home medications included metformin, sitagliptin and bisoprolol. His social history was significant for 15 pack years of cigarette smoking.

An initial assessment of his vital signs revealed hypoxia with oxygen saturation of 80 % at room air, heart rate of 84 beats per minute, and his respiratory rate was 20 per minute. A physical examination was unremarkable except for bilateral basal fine inspiratory crackles. Arterial blood gases showed mild respiratory alkalosis. An initial blood work-up showed hemoglobin of 8.7 g/dl, platelets count of 30×103/mm^3^, and white blood cell (WBC) count of 8.4×103/mm^3^. Prothrombin time (PT), partial thromboplastin time (PTT), international normalized ratio (INR), brain natriuretic peptides (BNP), and kidney and liver function test values were within normal limits. An electrocardiogram (ECG) and echocardiogram were normal. A chest radiograph showed bilateral interstitial infiltrates.

**Fig. 1:**
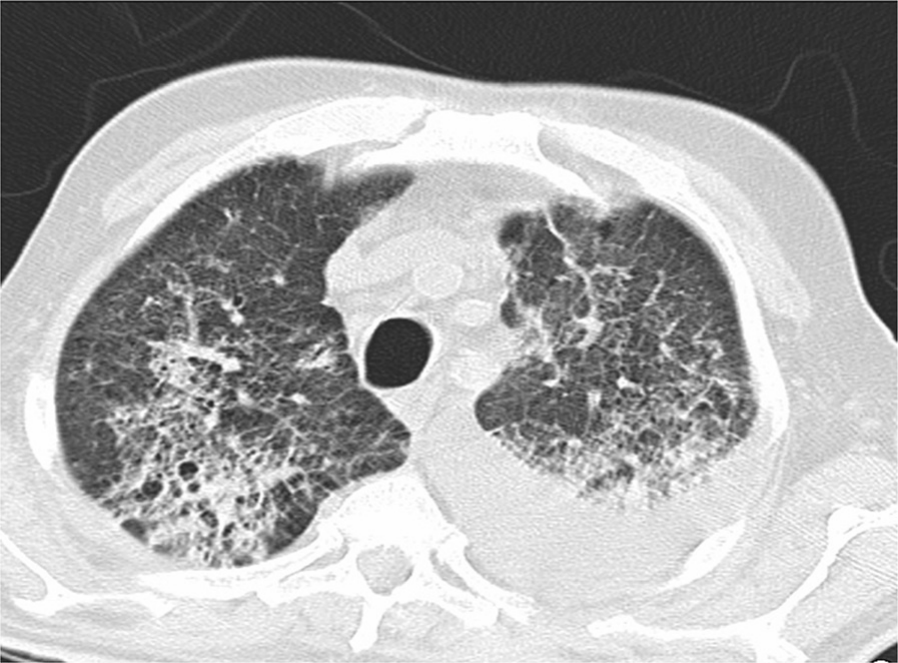
Computed tomography scan showing features of interstitial lung disease

He was started on dual antibiotics coverage (levofloxacin and piperacillin-tazobactam) for possible pneumonia. However, on the following day, his oxygen requirement increased dramatically; he was put on non-invasive ventilation and transferred to an intensive care unit (ICU). A non-contrast computed tomography (CT) scan of his chest was done at that time which revealed massive multifocal bilateral pulmonary consolidations, some of which were rounded with surrounding ground-glass opacities, as well as pleural effusion. A wide range of differential diagnoses was made at that time, including fungal, viral infection, and bronchiolitis obliterans with organizing pneumonia (BOOP) (Fig. 1).

Subsequently, his antibiotics were upgraded to caspofungin, teicoplanin, Tamiflu (oseltamivir phosphate), meropenem, and levofloxacin. A human immunodeficiency virus (HIV) test was performed and was reported as negative. Bronchoalveolar lavage and left-sided pleural fluid tapping was performed. Results of the lavage were negative for aerobic and anaerobic bacterial culture, fungal stain and culture, herpes simplex virus (HSV) and cytomegalovirus (CMV) polymerase chain reaction (PCR).

Analysis of his pleural fluid was consistent with a transudative pattern with a lactate dehydrogenase (LDH) value of 206 U/L (390 U/L in serum), protein 2.2 g/dl (5.3 in serum), glucose 210 mg/dl, and WBCs 100 with 90 % lymphocytes. His cytology was negative for malignant cells.

His condition did not improve until he was started on methylprednisolone 60 mg administered intravenously twice daily for suspected BOOP. After 7 days of methylprednisolone, his condition improved dramatically and he was successfully transferred to a general medical floor; during his stay at the general medical floor his dose of methylprednisolone was decreased to 40 mg administered intravenously twice daily for 4 days, then 20 mg administered intravenously twice daily for 3 days. He was then discharged home on home oxygen and oral prednisolone which was slowly tapered over 2 weeks.

A follow-up CT scan was performed 2 weeks later and showed a variable response in the previously mentioned multifocal pulmonary consolidations with a background of pulmonary interstitial thickening and ground-glass opacification involving both his lungs. Some foci had improved while others had progressed since the previous examination. In addition, a new opacity appeared on the lower lobe of his right lung with background interstitial thickening.

**Fig. 2:**
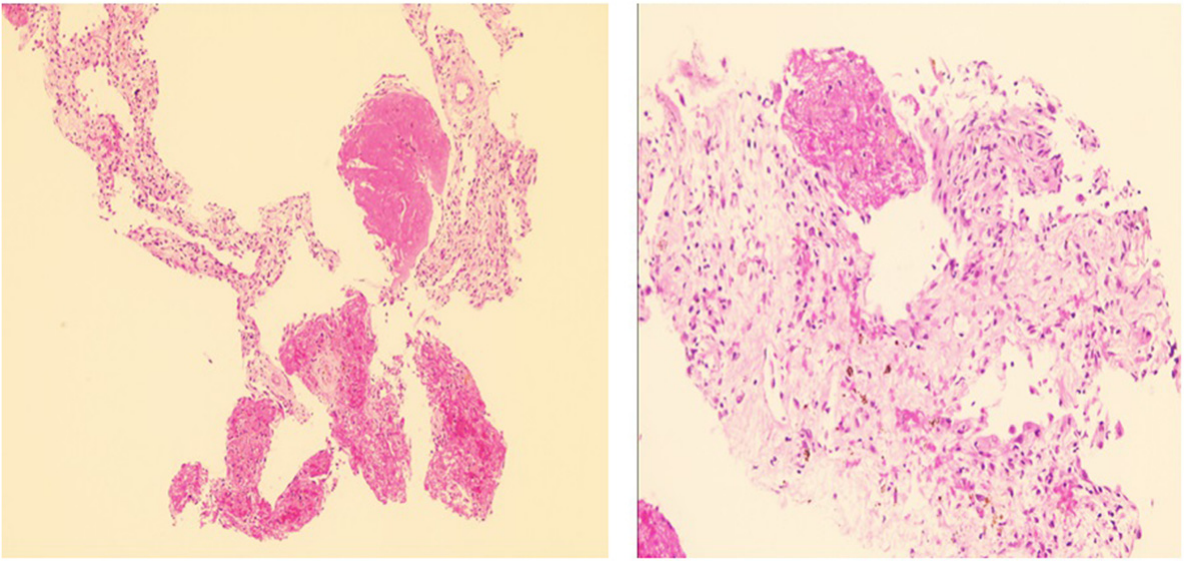
Biopsy which was consistent with cryptogenic organizing pneumonia

He was readmitted for a CT-guided Tru-Cut biopsy of the lower lobe of his right lung, which was performed 2 months later; the biopsy showed features of chronic nonspecific inflammation with macrophages consistent with organizing pneumonia (Fig. 2).

CMV and HSV immune stains were negative as well as Giemsa stain for fungal infections.

He was discharged home with oxygen support with a presumptive diagnosis of drug-induced ILD secondary to 5-azacitidine.

## Discussion

Prior to the introduction of hypomethylating agents, including azacitidine, as standard therapies for MDS, cytotoxic medications were the main stay of treatment. However, outcomes following these cytotoxic agents were disappointing. Furthermore, factors related to advanced age and comorbidities have limited the eligibility of most patients with MDS for bone marrow transplantation. Azacitidine is a cytidine analog which has a nitrogen atom instead of carbon at position five of the heterocyclic ring. Azacitidine undergoes phosphorylation to azacitidine triphosphate, which is incorporated into the ribonucleic acid (RNA), disrupting its metabolism and protein synthesis. Subsequently, azacitidine diphosphate is dephosphorylated into 5-aza-2'-deoxycytidine, which then undergoes phosphorylation into triphosphate and then binds to DNA methyltransferase inhibiting its action. Hypomethylation of CpG island is thought to accompany neoplastic transformation and silencing of the hypermethylated tumor suppressor gene *p15* ^*INK4B*^; this gene is found in MDS and is probably one of the most important genetic backgrounds in MDS which could be reversed by azacitidine [4].

Azacitidine has been associated with various adverse reactions including nausea, pyrexia, diarrhea, fatigue, cough, dyspnea, and bone marrow suppression which might result in febrile neutropenia, bleeding, and anemia. ILD is probably a rare adverse reaction secondary to azacitidine. A review of literature identified five previously reported cases of ILD in association with hypomethylating agents for treatment of MDS: four were reported with azacitidine [5–8] and one with decitabine [9]. We provide descriptions of clinical presentations and pulmonary outcomes for these reported cases in Table 1.

**Table 1:** Summary of the literature for cases of organizing pneumonia associated with the use of hypomethylating agents

Case report	Age/ Gender	Presentation	Onset	Radiographic findings	Lung biopsy findings	Treatment and outcome
Adams *et al*. [5]	71-year-old male	Shortness of breath and dyspnea	Immediately after 7-day course of first cycle of azacitidine	Bilateral perihilar airspace shadowing	Acute and chronic interstitial and alveolar fibrosis with chronic inflammation consistent with organizing pneumonia	Died few weeks later
Sekhri *et al*. [6]	56-year-old male	Not clear	Not clear	Non-resolving organizing pneumonia	Non-resolving organizing pneumonia pattern with bronchocentric granulomatous pattern suggestive of drug-induced lung disease	Successfully treated with steroids with resolution of lung infiltrate
Kuroda *et al*. [7]	72-year-old male	Pyrexia	After 7-day course of azacitidine	Acute interstitial pneumonitis	N/A	Died
Hueser and Patel [8]	55-year-old female	Hyperthermia	Not clear	Acute interstitial pneumonitis that led to acute respiratory failure	N/A	Responded to high-dose methylprednisolone
Vasu *et al*. [9]	65-year-old male	Cough, fever, and chills	2 weeks after initiating decitabine	Consolidation of lower lobe of left lung	Patchy areas of organizing pneumonia with fibrin balls within the alveoli and air spaces	Dramatic improvement with methylprednisolone
Current case	67-year-old male	Shortness of breath and dyspnea	2 weeks after second cycle of azacitidine	Multifocal pulmonary consolidation and bilateral interstitial thickening	Chronic nonspecific inflammation with macrophages consistent with organizing pneumonia	Variable response to steroids. Remained on home oxygen

*N/A* not available

In our case the Naranjo scale for adverse drug reaction was probable [10]. Gemcitabine which is another cytidine analog has been associated with lung toxicity as well [11].

We have discussed the case of a 67-year-old man diagnosed with MDS. He received two cycles of azacitidine administered intravenously (75 mg/m^2^, which was 150 mg administered intravenously) from days 1 to 7; he presented with dyspnea 2 weeks following the second cycle. His condition deteriorated significantly despite an early initiation of a wide spectrum of antibiotics. Failure to isolate a specific pathogen with bronchoalveolar lavage, as well as lack of fever, made an infectious etiology less likely.

Cryptogenic organizing pneumonia (COP) is one of the ILDs; the disease affects distal bronchioles, alveolar ducts, and alveoli. Although the pathogenesis is not fully understood, injury to alveolar epithelial cells in addition to imbalance between the activity of matrix metalloproteinase and its inhibitors seem to be the primary event that triggers COP. This leads to leakage of intracellular proteins which triggers an inflammatory reaction with recruitment of inflammatory cells, followed by production of fibromyxoid material resulting in plug formation against pores of Kohn. A high level of apoptosis is one of the striking features of this disease which makes it reversible in many cases [12, 13].

Our patient developed bilateral migratory lung opacities which is usually a striking feature of this disease [14]. The bronchoalveolar lavage as well as the biopsy failed to identify any specific infectious etiology. The biopsy from the new opacity at the zone of the lower lobe of his right lung was consistent with COP; 5-azacytidine was the only recent medication that he had received and as such it was the most likely culprit.

To the best of our knowledge this is the first case of azacitidine-induced COP that has occurred after a second cycle of azacitidine; our explanation for this was that our patient either developed a mild COP which was not apparent clinically or he maintained an immune response which sensitized him to the drug. After exposure to the second cycle these reactions became robust resulting in severe lung inflammation and damage. Furthermore this is the first reported case that has been associated with clinically significant pleural effusion that required tapping; although it is uncommon, pleural effusion can be associated with COP. A multicenter retrospective study of organizing pneumonia showed that 16 out of 74 patients who had either cryptogenic or secondary organizing pneumonia had pleural effusion [15]; de Gispert *et al*. described pleural effusion in two out of six patients who had organizing pneumonia [16]. Another retrospective study showed that four out of 18 patients with COP had pleural effusion [17]. The type of pleural effusion in those reports was not clear. In our case the patient developed bilateral pleural effusion that was greater on his left side. The analysis of the pleural fluid from his left side showed that it was transudative in nature. All of the following probably account for the transudative nature of his pleural effusion: the early lymphatic obstruction secondary to fibrotic changes in his lungs, the large quantity of intravenously administered fluid he received in the ICU as well on the general medical floor, and the tap that was done approximately 1 week after starting steroids which lessened the inflammatory reaction.

To the best of our knowledge, none of our patient’s regular medications have been associated with lung disease, although a case report was recently published on an association between oral hypoglycemic agents and intestinal lung disease, particularly metformin and glibenclamide [18]. However, our patient has been taking these drugs for many years, which made it highly unlikely that they contributed to the ILD.

## Conclusions

We concluded that there is a recognizable potentially life-threatening toxicity due to organizing pneumonia secondary to azacitidine in the setting of MDS treatment. This toxicity is not limited to the first cycle as in previous cases; furthermore, pleural effusion can be associated with this toxicity. Health care professionals should be aware of such toxicity as early recognition and timely management are critical to prevent permanent lung fibrosis.

## Consent

Written informed consent was obtained from the patient for publication of this case report and accompanying images. A copy of the written consent is available for review by the Editor-in-Chief of this journal.
